# Bridging clinic to home: domestic devices in dermatological diagnostics and treatments

**DOI:** 10.3389/fdgth.2025.1595484

**Published:** 2025-06-18

**Authors:** Diala Haykal, Frederic Flament

**Affiliations:** ^1^ Centre Laser Palaiseau, Private Practice, Cosmetic Dermatology, Palaiseau, France; ^2^ Research & Innovation, L’Oréal Research and Innovation, Clichy, France

**Keywords:** home-used devices, dermatology, diagnostics and therapy, regulatory challenges, IoT technologies

## Abstract

The integration of diagnostic and therapeutic tools into home-used devices has significantly transformed dermatology, making advanced skincare technologies more accessible to the public. Home-based diagnostic devices empower individuals to monitor, assess, and track skin conditions in real time, promoting earlier interventions and personalized skincare. Therapeutic devices, on the other hand, enable users to actively treat cosmetic and dermatological concerns, offering greater autonomy in managing skin health outside the clinical setting. These technologies, often inspired by clinical-grade equipment, promise enhanced patient engagement but also raise critical questions regarding safety, efficacy, and regulatory oversight. Importantly, the regulatory status of these devices, particularly for diagnostic tools, varies significantly across regions, affecting standards for quality, permitted energy outputs, and intended uses. This commentary separately explores the opportunities and challenges posed by home-used diagnostic and therapeutic devices, evaluates their roles in cosmetic dermatology, and highlights key insights from the literature to contextualize their growing influence on personalized skincare.

## Introduction

Technological innovation has long driven advancements in dermatology, with clinical-grade diagnostic and therapeutic tools playing a pivotal role in addressing skin concerns (1). Diagnostic tools, such as confocal microscopy and spectroscopy, are primarily designed to enable precise assessment, monitoring, and early detection of skin conditions. They provide detailed imaging and biochemical analysis that inform clinical decision-making and personalized care pathways. Therapeutic devices, including lasers, intense pulsed light (IPL), radiofrequency (RF) systems, and light-emitting diode (LED) technologies, are aimed at actively treating conditions, improving cosmetic outcomes, and promoting skin rejuvenation (2).

However, due to the high costs, technical complexity, and need for professional oversight, these tools have traditionally been confined to clinical environments. In recent years, the emergence of home-used devices that replicate these functions has introduced a new dimension to dermatological care (3). Consumers are now empowered to monitor their skin health and undertake certain treatments independently. Yet, this democratization of technology also raises concerns about efficacy, safety, and regulatory oversight. However, the shift from professional to personal use raises important questions about efficacy, safety, and regulatory oversight (3). The regulatory classification of diagnostic devices differs substantially between regions. For instance, the U.S. FDA and European regulatory bodies apply distinct standards for device approval, with further variability seen in Asian markets where energy thresholds and indications may be more permissive. These discrepancies necessitate careful consideration to ensure consistent quality and safety for users worldwide. This commentary explores the distinct roles and challenges of home-used diagnostic and therapeutic devices, emphasizing their implications for patient care, regulatory evolution, and the future of personalized dermatology.

## Diagnostic tools: From clinics to homes

Clinical diagnostic tools have long set the standard for precision in dermatology. Technologies such as confocal microscopy, Raman spectroscopy, and line-field confocal optical coherence tomography (LC-OCT) provide detailed imaging and biochemical analysis of the skin, enabling dermatologists to accurately diagnose conditions such as acne, pigmentation disorders, and aging (4–8). As Yun and Kwok have emphasized, these technologies rely on sophisticated light-based interactions with the skin, offering rich yet safe synergy with organic molecules (9). Despite their effectiveness, these tools remain largely inaccessible to the average consumer due to their high costs and the need for trained operators. The transition of diagnostic tools into home-used formats marks a significant shift in accessibility, with two distinct strategies emerging. The first approach prioritizes a highly detailed, comprehensive diagnosis performed by a dermatologist, followed by a more holistic, long-term monitoring strategy that considers context, movement, and lifestyle factors. The second strategy aims to achieve high diagnostic accuracy directly at home, reducing reliance on clinical visits. Smartphone-based multispectral imaging, as discussed by Kim et al., is a notable example of this trend. These systems enable users to analyze their skin's hydration, pigmentation, and elasticity in real time, leveraging mobile technology to bring diagnostic capabilities directly to consumers (10). While promising, such devices often fall short of clinical-grade systems in terms of resolution and diagnostic accuracy. Concerns regarding the transparency and validation of AI-driven diagnostic tools have emerged (11). Nevertheless, AI-powered skincare platforms, such as those evaluated by Reich et al., utilize advanced algorithms and sensor technologies to assess skin health and provide personalized recommendations (12). These platforms have the potential to transform skincare routines but rely heavily on user input and environmental factors, which can introduce variability in their performance. Muralidharan et al. have raised critical concerns about the transparency of AI-driven diagnostic tools, particularly regarding algorithm validation, data quality, and the reporting standards of FDA-approved devices and EADV task force guidelines (13, 14). For instance, a comprehensive review by Mineroff et al. examined the clinical applications of photobiomodulation, demonstrating that while some devices show promise, their efficacy depends on precise parameters such as wavelength, fluence, and duration (15).

Addressing these gaps is essential to fully realize the potential of home-used diagnostic tools.

## Treatment tools: bridging the clinical-home divide

Clinical therapeutic devices such as lasers, IPL systems, RF, and LED technologies have long been the cornerstone of dermatology for treating a wide range of conditions, including wrinkles, pigmentation and scars. These devices deliver targeted, high-energy treatments that require professional expertise to optimize outcomes and minimize risks (16). As Goldman has noted, such tools not only address aesthetic concerns, but also provide therapeutic benefits in order to offer comprehensive solutions for skin health (17). The emergence of home-used therapeutic devices has significantly expanded access to advanced skincare treatments. Devices such as LED masks, IPL tools and handheld lasers provide consumers with convenient, cost-effective options for addressing skin concerns (18). For instance, Ng et al. conducted a split-face pilot study on the efficacy of a home-use LED device at 637 and 854 nm for facial rejuvenation and demonstrated significant improvements in skin texture, fine lines, and overall skin tone on the treated side. Their study highlighted the potential of combined red and near-infrared light wavelengths to stimulate collagen production and enhance skin rejuvenation in a non-invasive, user-friendly manner (19).

Furthermore, Juhász et al. have extensively reviewed home-used IPL devices, demonstrating their effectiveness for hair removal and pigmentation correction (20). However, their findings also underscore the importance of consistent use, as these devices often require longer treatment durations to achieve results comparable to professional systems. Hession et al. have highlighted the growing popularity of handheld laser devices, which offer portability and ease of use (21). More specific studies, such as that conducted by Gold et al., evaluate self-applied blue light therapy for acne as significantly effective in acne lesion reduction (22). However, they emphasized the importance of adherence to treatment protocols, noting that improper use or a lack of professional oversight could limit the effectiveness of these devices. While these devices provide a viable option for addressing localized skin issues, their reduced power levels often result in slower progress compared to clinical treatments (23). These findings illustrate the trade-offs involved in designing home-use devices that prioritize safety for unsupervised use.

## Opportunities and challenges in diagnostic and treatment devices

The proliferation of home-used diagnostic and therapeutic devices represents a paradigm shift in dermatology, offering unprecedented accessibility and convenience (24, 25). By enabling consumers to monitor and treat their skin at home, these devices have democratized skincare, fostering greater engagement and awareness (26–29). The integration of AI and internet of things (IoT) technologies further enhances their potential, allowing for personalized treatment plans and real-time monitoring (30). As Bu et al. have noted, AI-driven systems can adapt treatment parameters to individual skin profiles, while IoT connectivity allows devices to sync with apps for progress tracking and remote consultations (18). These advancements hold the promise of transforming domestic devices into powerful tools for personalized, data-driven skincare. However, efficacy and safety concerns are among the most pressing issues remaining a challenge. As Hattersley et al. have documented, user-reported complications such as burns and blisters highlight the risks associated with improper use of these device (31). Reduced power levels, while ensuring safety, often compromise efficacy, requiring longer treatment durations and greater user compliance. User adherence, as Gold et al. have noted, remains a critical barrier to achieving optimal outcomes, underscoring the importance of consumer education and structured support systems (22). Given their increasing use, home-based devices raise substantial regulatory challenges that must be addressed to ensure safety, efficacy, and equitable access across regions. In the United States, devices intended for home use must often meet strict FDA requirements regarding safety and intended use, while in Europe, CE marking is required but categorization may differ based on the device's risk level under the Medical Device Regulation (MDR). Furthermore, permitted power levels, intended indications, and required instructions for use may differ across regions. In some countries, particularly in parts of Asia, regulatory standards for energy-based devices can be more permissive, especially concerning allowable energy outputs and claims for cosmetic indications (13, 14, 32). This disparity highlights the need for international collaboration and global harmonization efforts to establish consistent safety and efficacy standards. Without such frameworks, variability in product quality, performance, and user outcomes can undermine consumer trust and limit the full integration of home-used devices into dermatological care. Addressing these regulatory and practical challenges will be crucial for ensuring the safe, effective, and equitable use of these rapidly expanding technologies.

## Global access and affordability

The integration of at-home devices into formal healthcare systems represents an untapped opportunity to enhance dermatologist-patient collaboration (33). Additionally, they have the potential to address disparities in dermatological care, particularly in underserved or remote areas where access to clinics may be limited. In regions with limited healthcare infrastructure, these tools can empower individuals to monitor and manage their skin health without the need for frequent clinical visits (34–36). Smart diagnostic apps, for example, allow users to track skin conditions and seek early interventions, potentially reducing the burden on healthcare systems. However, affordability remains a key concern. While marked as cost-effective alternatives to clinical treatments, many home-use devices require a significant upfront investment, which can be prohibitive for consumers, especially in low-income regions. Additionally, some devices rely on consumables, such as replacement cartridges for IPL systems, increasing their long-term cost. As a result, whether these tools truly democratize dermatological care or primarily cater to wealthier markets remains a subject of debate (37, 38). However, certain compact, accurate, and relatively affordable tools integrated into smartphone ecosystems could help bridge this accessibility gap. For instance, the GPSkin, a validated device for measuring hydration and transepidermal water loss (TEWL), is available for approximately $250 and can synchronize with a smartphone. Such innovations demonstrate how connected dermatological monitoring can provide reliable data at a relatively accessible price point, potentially expanding the reach of at-home skin health management (39). To further narrow this accessibility gap, telepresence-guided consultations could serve as a key innovation. By enabling dermatologists to remotely guide patients through the use of diagnostic and therapeutic devices, telemedicine combined with smart home devices could significantly enhance the accuracy and safety of at-home treatments. This approach would be particularly beneficial and relevant for patients using advanced skincare technologies like home-use lasers, light-based devices, and RF tools, where proper application and real-time adjustments are crucial for both efficacy and safety.

## Integration into healthcare systems

The main aspect concerning the integration of home-used devices into formal healthcare systems lies within dermatologist-patient collaboration. By allowing patients to monitor their skin health at home, these devices can provide valuable data for dermatologists to review during consultations (40). For example, smartphone-based diagnostic tools can track the progression of skin conditions over time, offering a comprehensive picture that aids treatment planning (41, 42). In the same way, telemedicine platforms can complement these devices by enabling remote consultations. Dermatologists can use data collected from home-used tools to assess patients’ conditions and adjust treatment plans without requiring in-person visits (43). This hybrid model of care not only improves access, but also enhances patient engagement by fostering a sense of ownership over their skincare routines. To realize this potential, interoperability between home-used devices and clinical systems must be prioritized. Standardized protocols for data sharing and analysis can ensure that the information generated by these devices is compatible with existing healthcare infrastructure (44). Collaboration between manufacturers, healthcare providers, and regulatory bodies will be key to creating an ecosystem where home-used devices complement professional care seamlessly.

## Impact of consumer behavior and trends

The increasing demand for personalized and convenient skincare has been a major driver behind the development and adoption of home-used dermatological devices (45). Consumers today are more informed about skincare, largely due to the influence of social media and the proliferation of online communities that discuss and promote various skincare routines and products (46). The abundance of dermatological content has created a market for tools that allow individuals to take control of their skincare. Home-used devices, such as smartphone-based diagnostic tools or LED therapy masks are positioned as accessible and user-friendly solutions for addressing a variety of skin concerns (19). At the same time, social media plays a pivotal role in popularizing these devices. Platforms like Instagram, TikTok, and YouTube feature content creators who demonstrate the use of home-used tools, often providing testimonials that boost consumer confidence (47). However, these trends can sometimes lead to inflated expectations (48). The effectiveness of these devices varies significantly based on the condition being treated, user adherence and the device's design. This dichotomy underscores the need for consumer education and proactive fight against misinformation to balance the enthusiasm driven by marketing with realistic expectations about outcomes.

## Comparison with professional treatments

Despite making advanced dermatological care more accessible, home-used devices cannot fully replicate the precision and efficacy of professional treatments. Clinical-grade diagnostic tools, such as confocal microscopy and spectroscopy provide unmatched detail and accuracy, thus enabling dermatologists to diagnose and treat complex conditions. Similarly, professional therapeutic devices like high-power lasers and advanced energy-based devices offer results that are difficult to achieve with at-home alternatives. Home-used devices are most effective for routine maintenance and addressing mild cosmetic concerns (3, 18). For instance, devices such as IPL hair removal systems or LED masks for acne and anti-aging provide measurable benefits for users who are consistent with their treatments (23). However, for severe conditions like deep scarring, complex pigmentation disorders, or medical-grade wrinkle reduction, professional interventions remain the gold standard (49). Emerging research suggests that some innovations in home-use devices may help bridge this gap. For example, advancements in micro-needling technology, particularly when combined with targeted cosmetic formulations (50, 51). Thus, home-used devices would be considered as complementary tools (3). They can extend the longevity of professional treatments by allowing users to maintain results between clinic visits; but they cannot substitute clinical treatments. Subsequently, the balance of professional and personal care reflects an emerging hybrid model in dermatology, where patients can engage with both clinical and home-based tools for comprehensive skin health management. The study performed by Bu et al. underscores the growing role of home-used devices as complementary tools in dermatological care. While these devices are effective for routine maintenance and mild cosmetic concerns, they cannot match the precision and efficacy of clinical-grade tools and professional treatments (18). By bridging professional and at-home care, this study highlights an emerging hybrid model, enabling patients to maintain results and engage in comprehensive skin health management.

## The role of dermatologists: guiding safe use of home devices

As at-home dermatology devices continue to gain popularity, dermatologists play a pivotal role in ensuring their safe and effective use. The increasing availability of these tools, ranging from LED masks to at-home IPL and RF devices, empowers consumers to take a more active role in their skincare (3). However, the clinical effectiveness and safety of these devices remain highly variable, necessitating expert guidance. Studies indicate that when dermatologists provide structured education on proper device selection, realistic treatment expectations, and correct usage, patients demonstrate improved adherence and achieve better outcomes (15, 18). Misuse or overuse of these devices can lead to complications such as burns, post-inflammatory hyperpigmentation, and suboptimal results, particularly in individuals with darker skin tones or sensitive skin. As previously discussed, regulatory oversight remains critical to ensure the safe use of at-home technologies (52). Integrating home-use devices into professional dermatologic care presents opportunities to enhance treatment continuity. For example, dermatologists can recommend specific devices for post-procedure maintenance, collagen stimulation, or acne management, ensuring they complement clinical interventions rather than replace them (3). By actively participating in patient education and regulatory discussions, dermatologists can bridge the gap between consumer convenience and clinically validated dermatologic care, optimizing both safety and long-term skin health.

## Enhancing clinical relevance and addressing safety considerations

At-home dermatology devices have expanded access to skincare technologies, but their clinical effectiveness, safety, and real-world outcomes remain key considerations (53). While these devices, such as LED masks, IPL, and RF tools, offer convenience, their efficacy is limited by lower energy output, lack of professional calibration, and variable user compliance, often resulting in more gradual and less predictable results compared to in-office treatments. As previously discussed, regulatory oversight remains a key pillar; in this section, we focus specifically on user adherence and safety considerations that influence the clinical effectiveness of home-used devices.

Many studies evaluating these technologies are constrained by small sample sizes, short follow-ups, and limited statistical significance, necessitating a critical approach to interpreting their clinical relevance. Safety concerns are particularly pronounced in darker skin tones, where at-home IPL devices may increase the risk of post-inflammatory hyperpigmentation, and at-home microneedling carries risks of infection, scarring, and excessive trauma if misused. Unlike professional treatments, home-use devices often lack regulatory oversight, making product quality and safety inconsistent (54). Despite these limitations, they may serve as valuable adjuncts to clinical treatments, supporting post-procedure recovery, maintaining collagen stimulation, and prolonging the benefits of in-office care. Dermatologists play a crucial role in guiding patients toward evidence-based use, ensuring appropriate device selection, proper application, and realistic expectations to minimize risks while optimizing results (55, 56). As the industry evolves, integrating AI-driven personalization and improved regulatory standards will be essential in bridging the gap between consumer convenience and clinically validated dermatologic care.

## Ethical and privacy concerns

The integration of AI and IoT technologies into home-used devices has brought significant advancements in personalization and functionality, but it has also raised ethical and privacy concerns (57, 58). Many of these devices collect sensitive data, including images of users’ skin and detailed health metrics, which are processed and stored to provide personalized recommendations. This raises questions about data security, ownership and the potential misuse of such information. Transparency in how companies collect, store and use data is crucial to building consumer trust (59). Users should be informed about how their information is protected, and whether it will be shared with third parties. Additionally, companies must adhere to robust data protection regulations, such as the General Data Protection Regulation (GDPR) in Europe, to ensure compliance with privacy standards. Another ethical concern lies in the potential biases embedded within AI algorithms (60). If the training data for these systems does not adequately represent diverse skin types, the resulting recommendations may be less effective or even inaccurate for certain populations (61). Ensuring diversity in algorithm training and testing is essential to make these devices inclusive and equitable.

## Long-term studies and evidence gaps

Despite the growing popularity of home-used devices, there is a lack of long-term studies evaluating their safety and efficacy. Most existing research focuses on short-term outcomes, leaving unanswered questions about the cumulative effects of prolonged use. For example, repeated use of certain light-based therapies may have unknown implications for skin health over time. Additionally, user compliance plays a crucial role in determining long-term efficacy. Many treatments, such as those for acne or pigmentation, require consistent use, and adherence varies significantly among users. For instance, when treating hyperpigmentation, initial improvements achieved with home-used light-based devices could be compromised if users do not maintain strict photoprotection. Research has shown that standardized daily sunscreen applications can significantly slow the progression of pigmentation and wrinkle formation. A recent prospective randomized trial demonstrated that, compared to a classical skincare routine without strict photoprotection, daily sunscreen use over one year led to visible improvements in pigmentation irregularities and fine lines across skin phototypes II to VI (62). Thus, integrating photoprotective measures as part of a structured home treatment regimen may enhance and prolong clinical benefits. Future randomized controlled trials are needed not only to assess clinical outcomes and potential adverse effects but also to evaluate adherence, user satisfaction, and strategies to improve compliance. Comparative studies between home-used devices and professional treatments would further clarify their relative strengths and limitations. Addressing these evidence gaps is essential to guide both consumers and healthcare professionals in making informed decisions and to establish regulatory frameworks that prioritize both safety and efficacy.

## Future directions: From continuous monitoring to molecular diagnostics

The evolution of home-used dermatological devices is moving beyond periodic self-assessment toward continuous, real-time skin monitoring through wearable technologies. Miniaturized diagnostic tools embedded in rings, watches, patches, or even fabrics could offer constant evaluation of key skin parameters such as hydration, elasticity, temperature, and even early inflammatory changes. By integrating sensors directly into devices worn daily, users and dermatologists could obtain dynamic skin health profiles, allowing for earlier intervention and personalized preventive strategies.

Moreover, the next frontier lies in coupling these technologies with molecular diagnostics, particularly in the field of epigenetics. Non-invasive or minimally invasive sampling of skin biomarkers could reveal underlying epigenetic modifications associated with aging, environmental exposures, or disease states. Such information would offer a deeper, more predictive understanding of skin health, transcending visual inspection to reach the molecular and functional level. By integrating wearable monitoring and epigenetic insights, dermatological care could become not only more personalized but also more preventive, anticipating changes before clinical symptoms appear. This approach would further reinforce the dermatologist's role at the center of an intelligent, continuously connected skincare ecosystem.

## Personal perspectives and future directions

Based on the current landscape, a hybrid model appears to be the most promising approach for the future of dermatological care. In this model, home-used diagnostic and therapeutic devices would not function independently, but would be fully integrated into a connected ecosystem centered around the expertise of dermatologists. Patients would use wearable and home-based technologies to continuously monitor key skin parameters, while dermatologists would curate, interpret, and personalize the collected data to dynamically adapt preventive and therapeutic strategies. The success of this model will depend heavily on achieving interoperability between home devices, mobile applications, and clinical systems, creating a seamless and efficient continuum of care.

In the next five to ten years, we anticipate a progressive convergence between wearable diagnostics, AI-driven skin analysis, and targeted home therapies, enabling real-time, dynamic skin health management. Continuous data collection through miniaturized technologies, such as rings, watches, or adhesive patches, combined with molecular insights from non-invasive epigenetic monitoring, will likely open new dimensions in personalized dermatology. Dermatologists will increasingly shift from episodic treatment providers to ongoing skin health coaches, guiding patients through highly individualized care trajectories supported by connected technologies.

Given the growing complexity and influence of these tools, regulatory frameworks will need to evolve accordingly. Leadership should not rest solely on governmental agencies but rather involve dermatological societies, independent academic researchers, international regulatory bodies, and the industry. Creating globally recognized standards for clinical validation, ensuring algorithm transparency and bias mitigation, and enforcing safety standards that account for diverse skin types will be essential. Dermatologists must be positioned at the center of this regulatory evolution to ensure that technological innovations remain clinically meaningful, ethically grounded, and patient centered. Only through such an integrated approach can the full potential of home-used dermatological technologies be safely and effectively realized.

## Conclusion: toward balanced integration

Home-used diagnostic and therapeutic devices are reshaping the landscape of dermatology, making advanced skincare technologies more accessible than ever before. While these innovations hold significant promise, their limitations in efficacy, safety, and regulation underscore the importance of viewing them as complementary to professional care. Dermatologists play a crucial role in guiding consumers toward safe and effective use of these devices, through bridging the gap between clinical and personal care. Beyond individual devices, what is truly emerging is a connected ecosystem where diagnostic tools, therapeutic devices, AI algorithms, and telemedicine platforms interact seamlessly. In this model, the dermatologist remains at the core, acting as an expert coach who interprets data, tailors treatment pathways, and ensures the highest standards of safety and efficacy. The future of home-used devices lies in their ability to integrate into this broader professional ecosystem, allowing for hyper-personalized, real-time skincare management. By fostering collaboration among manufacturers, regulators, and healthcare professionals, the field of dermatology can fully embrace the potential of these tools while maintaining high standards of clinical relevance and patient-centered care.

## Data Availability

The original contributions presented in the study are included in the article/Supplementary Material, further inquiries can be directed to the corresponding author.
